# Searching for Monogenic Autoimmune Etiology in Patients With Type 1 Diabetes Onset Before 30 Months of Age

**DOI:** 10.1210/clinem/dgaf049

**Published:** 2025-01-28

**Authors:** Laura Saso-Jiménez, Inés Urrutia, Begona Calvo, José Ramón Bilbao, Ana Lucía Gómez-Gila, Isabel Leiva-Gea, Andrea Jiménez-Sanchis, Itxaso Rica, Luis Castano, Rosa Martínez

**Affiliations:** Endocrinology and Diabetes Research Group, Biobizkaia Health Research Institute, 48903 Barakaldo, Spain; UPV/EHU, CIBERDEM, CIBERER, Endo-ERN, 48903 Barakaldo, Spain; Endocrinology and Diabetes Research Group, Biobizkaia Health Research Institute, 48903 Barakaldo, Spain; UPV/EHU, CIBERDEM, CIBERER, Endo-ERN, 48903 Barakaldo, Spain; Endocrinology and Diabetes Research Group, Biobizkaia Health Research Institute, 48903 Barakaldo, Spain; Department of Medical Oncology, Hospital Universitario Cruces, 48903 Barakaldo, Spain; Endocrinology and Diabetes Research Group, Biobizkaia Health Research Institute, 48903 Barakaldo, Spain; UPV/EHU, CIBERDEM, CIBERER, Endo-ERN, 48903 Barakaldo, Spain; Pediatric Endocrinology Unit, Hospital Universitario Virgen del Rocío, 41013 Sevilla, Spain; Pediatric Endocrinology Unit, Hospital Materno Infantil Regional de Málaga, 29011 Málaga, Spain; Biomedical Research Institute of Malaga (IBIMA), 29590 Málaga, Spain; Department of Pharmacology and Pediatrics, Malaga University (UMA), 29010 Málaga, Spain; Endocrinology and Diabetes Research Group, Biobizkaia Health Research Institute, 48903 Barakaldo, Spain; Endocrinology and Diabetes Research Group, Biobizkaia Health Research Institute, 48903 Barakaldo, Spain; UPV/EHU, CIBERDEM, CIBERER, Endo-ERN, 48903 Barakaldo, Spain; Pediatric Endocrinology Unit, Hospital Universitario Cruces, 48903 Barakaldo, Spain; Endocrinology and Diabetes Research Group, Biobizkaia Health Research Institute, 48903 Barakaldo, Spain; UPV/EHU, CIBERDEM, CIBERER, Endo-ERN, 48903 Barakaldo, Spain; Endocrinology and Diabetes Research Group, Biobizkaia Health Research Institute, 48903 Barakaldo, Spain; UPV/EHU, CIBERDEM, CIBERER, Endo-ERN, 48903 Barakaldo, Spain

**Keywords:** monogenic autoimmune diabetes, type 1 diabetes, early-onset, pediatrics

## Abstract

**Introduction:**

The most frequent form of diabetes in pediatric patients is polygenic autoimmune diabetes (type 1 diabetes [T1D]), but single-gene variants responsible for autoimmune diabetes have also been described. Both disorders share clinical features, which can lead to monogenic forms being misdiagnosed as T1D. However, correct diagnosis is crucial for therapeutic choice, prognosis, and genetic counseling. The aim of this study was to search for monogenic autoimmune diabetes in Spanish pediatric patients with early-onset T1D.

**Methods:**

Among 500 Spanish pediatric patients with T1D, those with disease onset between 9 and 30 months of age were selected for screening for monogenic autoimmune diabetes (n = 44). Genetic testing was performed by next-generation sequencing with a customized panel that included the major causative genes for monogenic autoimmune syndromes, including early-onset diabetes: *AIRE*, *CTLA4*, *FOXP3*, *IL2RA*, *ITCH*, *LRBA*, *STAT1*, *STAT3*, *STAT5B*. RT-PCR and cDNA sequencing of the RNA isolated from whole blood were used to analyze splicing variants.

**Results:**

Genetic screening identified, in 2 patients with diabetes onset before 1 year of age, 2 likely pathogenic novel variants affecting canonical splicing sites: c.286-12_290del in *STAT5B* and c.-22-2delA in *FOXP3*. RNA analyses demonstrated that both variants modify mRNA splicing. The variant in *STAT5B* induced exon 4 skipping and the variant in *FOXP3* caused a deletion of 16 nucleotides before the transcription start site.

**Conclusion:**

T1D onset in the first year of life may indicate monogenic autoimmune diabetes and molecular testing may be recommended.

Type 1 diabetes mellitus (T1D) is a chronic disease caused by progressive autoimmune destruction of insulin-producing pancreatic β cells, leading to lifelong dependence on exogenous insulin. It can be diagnosed at any age, but peaks in presentation occur between 5 and 7 years of age and at or near puberty (1). The etiology of the disorder is not completely understood, but evidence suggests that a polygenic predisposition (HLA and non-HLA genes) together with environmental triggers play a decisive role in the initiation of the autoimmune process. Autoantibodies targeting several pancreatic islet molecules such as insulin autoantibodies (IAA), glutamate decarboxylase autoantibodies (GADA), islet tyrosine phosphatase 2 autoantibodies (IA2A), and zinc transporter-8 autoantibodies (ZnT8A) are the main predictive and diagnostic biomarkers for the disease (2).

In contrast to polygenic diabetes, monogenic diabetes is caused by defects in a single gene with an autosomal dominant or recessive inheritance, and accounts for up to 4% of all cases of diabetes diagnosed before the age of 30 years (3). A key feature for identifying this type of diabetes is the absence of autoimmunity. However, single gene variants that are responsible for multiautoimmune syndromes that include diabetes have also been described (4). In recent years, advances in genomics have led to the identification of at least 9 genes that cause monogenic autoimmune syndromes with early-onset diabetes: *AIRE, CTLA4, FOXP3, IL2RA, ITCH, LRBA, STAT1, STAT3,* and *STAT5B* (5-8). These monogenic autoimmune forms of diabetes represent a fairly rare condition in pediatrics that shares basic features with T1D such as early diabetes onset associated with the presence of circulating pancreatic islet autoantibodies and requirement of insulin therapy (6, 9). Because T1D is the most common type of diabetes in pediatrics, most cases of monogenic autoimmune diabetes are likely to be misdiagnosed as T1D. Moreover, the clinical manifestations may be highly heterogeneous and is not always accompanied at onset by autoimmune disorders other than diabetes (10-12). Despite this, in the few studies published so far, the search for unrecognized monogenic autoimmune diabetes is mainly focused on patients with other autoimmune conditions in addition of T1D (6, 9). For this reason, this study proposes a different approach to screen for monogenic autoimmune diabetes, focused on pediatric patients with an unusual early onset of T1D (between 9 and 30 months of age), regardless of whether they have other autoimmune diseases.

## Methods

### Study Cohort

We studied 500 unrelated pediatric patients with recent onset T1D diagnosed according to the International Society for Pediatric and Adolescent Diabetes criteria (13). Participants were recruited from 7 hospitals in Spain between 2012 and 2021 (age range, 9 months to 14 years; 53% males). Baseline characteristics of the cohort are described elsewhere (14). Pancreatic autoantibodies (IAA, GADA, IA2A, and ZnT8A) were determined in serum at diagnosis. Autoimmunity was defined as the presence of at least 1 positive autoantibody.

In total, 44 children with T1D onset between 9 and 30 months of age and at least 1 positive autoantibody were selected for the study, regardless of the presence of other autoimmune diseases. Patients with neonatal diabetes up to 9 months of age (15) were outside the objective of this study. At diabetes onset, clinical data such as age, family history of diabetes, presence of diabetic ketoacidosis (DKA) according to International Society for Pediatric and Adolescent Diabetes (16) and HLA-DRB1 genotype were collected (Table 1). In patients with a likely pathogenic alteration, more detailed clinical data recorded at diagnosis and at the last follow-up were added (Table 2).

**Table 1.** Baseline characteristics of the cohort selected for genetic study

	T1D onset (9-30 mo)
Sex (males)	22/44 (50%)
Age at DM onset (mo)	22.5 (15.2-25.7)
First-degree relatives with any DM	9/44 (20%)
DKA at diagnosis	23/42 (55%)
Positive autoimmunity	
IAA	40/42 (95%)
GADA	29/44 (66%)
IA2A	21/44 (48%)
ZnT8A	8/44 (18%)
Multiple autoantibodies	34/44 (77%)
HLA-DRB1 genotype	
High risk	21/44 (48%)
Moderate risk	21/44 (48%)
Low risk	2/44 (4%)

Age at diagnosis is shown as median and interquartile range (interquartile range, P_25_-P_75_). The remainder data are shown as n/total (%). Multiple autoantibodies: more than 1 positive autoantibody. High risk (DR3/DR4), moderate risk (DR3/DR3, DR4/DR4, DR3/DRX, DR4/DRX), low risk (DRX/DRX). DRX corresponds to any HLA-DRB1 allele different from DR3 and DR4.

Abbreviations: DM, diabetes mellitus; DKA, diabetic ketoacidosis; IAA, insulin autoantibodies; GADA, glutamate decarboxylase autoantibodies; IA2A, islet tyrosine phosphatase 2 autoantibodies; T1D, type 1 diabetes; ZnT8A, zinc transporter 8 autoantibodies.

**Table 2.** Clinical characteristics of the patients with suspected monogenic autoimmune diabetes

Disease onset data	Patient 1	Patient 2
Sex	Male	Male
Positive antibodies	IAA, GADA, IA2A	IAA
HLA-DRB1 genotype	DR3/DR3	DR3/DR4
Age of diabetes onset	12 mo	9 mo
DKA	Moderate	Moderate
Blood glucose (mmol/L)	28.5	18.3
HbA1c (mmol/mol)	85	73
Insulin dose (UI/K/day)	0.55	0.76
Insulin therapy	MDI	MDI
**Last revision**	**Patient 1**	**Patient 2**
Time of evolution (y)	7.5	6
Insulin dose (UI/K/day)	0.63	0.99
HbA1c (mmol/mol)	52	48
Autoimmune diseases	Diabetes	Diabetes

Abbreviations: DM, diabetes mellitus; DKA, diabetic ketoacidosis; GADA, glutamate decarboxylase autoantibodies; IAA, insulin autoantibodies; IA2A, islet tyrosine phosphatase 2 autoantibodies; MDI, multiple daily injection.

This research was carried out in accordance with the Declaration of Helsinki (2008) of the World Medical Association. The study was approved by our local ethics committee, CEIm-E (Comité de Ética de la Investigación con medicamentos de Euskadi), and informed consent was obtained from all participants and/or their legal guardians.

### Autoantibody Analyses

Pancreatic autoantibodies (IAA, GADA, IA2A, and ZnT8A) were determined in serum by standardized radioimmunoassays. IAA was determined using a competitive fluid-phase radioassay that uses ^125^I-labelled recombinant human insulin as antigen (17). In brief, serum samples were incubated in duplicated with ^125^I-insulin at 4 °C for 1 week in the absence and presence of competition with nonradioactive insulin. The immune complexes were precipitated with polyethylene glycol and the amount of precipitated radioactivity was determined in a gamma radiation counter. Specificity was 98% to 100%, with a sensitivity of 60% to 65% (internal validation, past 5 years).

GADA, IA2A, and ZnT8A radioimmunoassays followed the same standard protocol, using 3 different ^35^S-methionine-labeled recombinant human antigens: full-length glutamic acid decarboxylase (GAD65), the intracellular domain (amino acids 605-979) of islet antigen 2 (IA2ic), and a hybrid construct of the ZnT8 COOH-terminal domain (amino acids 265-369) containing Arg325 and Trp325 variants (18-20). Labeled antigens were produced by in vitro transcription/translation using the TNT SP6 Coupled Reticulocyte Lysate System (Promega, Southampton, UK). The cDNA clones pEX9, pIA2ic, and pJH5.2 were kindly provided by Å. Lernmark (University of Washington, Seattle, WA, USA), E. Bonifacio (San Raffaele Scientific Institute, University of Milan, Italy), and J. Hutton (Barbara Davis Center, University of Colorado, Denver, Com USA), respectively. Briefly, serum samples were incubated with either radiolabeled antigen at 4 °C overnight. Antibody-bound antigens were subsequently precipitated using Protein A Sepharose beads, washed, and recovered by filtration. The amount of bound radiolabeled antigen was quantified in a β radiation counter. Specificity was 99% to 100% for all 3 autoantibody assays and sensitivity was 68% to 88% for GADA, 62% to 78% for IA2A, and 62% to 74% for ZnT8A (Islet Autoantibody Standarization Program, past 5 years).

### HLA-DRB1 genotyping

HLA-DRB1 genotyping was performed using PCR-SSO with Luminex technology, employing the LABType RSSOH2B1 (HLA-DRB1-HD) commercial kit (One Lambda, Inc., Canoga Park, CA, USA). All procedures adhered to the manufacturer's protocols. HLA-DRB1 risk alleles for autoimmune diabetes were defined in a previous report (21). HLA-DRB1 genotypes were categorized as high risk (DR3/DR4), moderate risk (DR3/DR3, DR4/DR4, DR3/DRX, DR4/DRX), and low risk (DRX/DRX). DRX corresponds to any HLA-DRB1 allele other than DR3 and DR4, including DRB1*0403.

### Genetic Analyses

DNA extraction from peripheral blood was performed using the MagPurix Blood DNA Extraction Kit (Zinexts Life Science Corp., New Taipei City, Taiwan, ROC). DNA quality and quantification was assessed by Nanodrop and Qubit 2.0 Fluorometer (Thermo Fisher Scientific, Waltham, MA, USA) following the manufacturers’ instructions.

#### Monogenic autoimmune diabetes screening

Genetic testing was performed by next-generation sequencing (NGS) with a customized gene panel using Ion Ampliseq sequencing technology (Thermo Fisher Scientific). The gene panel comprised the 5′ and 3′ untranslated regions, promoters, the entire coding region, and exon-intron boundaries (±50 bp) of 69 genes related with carbohydrate metabolism disorders, including the main genes associated with a monogenic form of autoimmune diabetes: *AIRE, CTLA4, FOXP3, IL2RA, ITCH, LRBA, STAT1, STAT3,* and *STAT5B*. Libraries were prepared using the Ion Ampliseq Library Kit v2.0 according to manufacturer's instructions. Sequencing was performed on the Ion GeneStudio S5 System. For base calling, read filtering, alignment to the reference human genome GRCh38, and variant calling, the Ion Torrent Suite and the Ion Reporter Softwares were used. Variants with minor allele frequency (MAF) > 0.01 in population databases (1000 Genomes Project, GnomAD, and dbSNP) were excluded. Variants of interest were confirmed by Sanger sequencing. When possible, parents and other family members were analyzed.

#### Classification of variants

Variants were classified according to the American College of Medical Genetics and Genomics and the Association for Molecular Pathology guidelines (22). In brief, classification was based on the strength of available evidence, which includes allele frequency in population databases, the type of variant (eg, frameshift, nonsense and essential splice variants), the localization in the protein domain (a mutational hot spot and/or critical and well-established functional domain) and the clinical, functional, and genotype-phenotype data from the literature and disease databases (Human Gene Mutation Database Professional, ClinVar, PubMed). If such variants had not been reported previously, they were evaluated to predict their possible functional significance using in silico prediction tools for missense variants (SIFT, PolyPhen2, PROVEAN, Mutation Taster, Panther, MutPred2, and SNPs&GO) and for splicing variant (Varseak, Human Splicng Finder, NetGene2). Rare variants were considered of uncertain significance (VUS) if the available information had limited or contradictory evidence.

#### Study of mRNA

RT-PCR and sequencing were performed to assess the effect of splice site variants. Total RNA was extracted from patients and progenitors from whole blood collected in Tempus tubes using GeneJET Stabilized and Fresh Whole Blood RNA kit. The cDNA was synthesized using RevertAid First Strand cDNA Synthesis kit (Thermo Fisher Scientific) and then amplified with specific primers designed in exon-exon boundaries. Thus, in the case of the *STAT5B* gene, the forward primer spanned the junction of exons 1 and 2 and the reverse primer that of exons 5 and 6. For *FOXP3* gene, the forward primer annealed to noncoding exon 1 and the reverse primer spanned the junction of exons 3 and 4. Amplified PCR products were separated, extracted from a 2% agarose gel, and sequenced by Sanger to check for a possible alternative splicing pattern.

## Results

In total, 44 patients with diabetes onset between 9 and 30 months of age were selected for genetic testing to rule out a monogenic form of autoimmune diabetes. The median diabetes onset age in the final cohort was 22 months (interquartile range, 15-25) and all 44 selected patients were positive for at least 1 pancreatic autoantibody. Specifically, 22.7% (10/44) had 1 positive autoantibody, 40.9% (18/44) had 2, 25% (11/44) had 3, and 11.4% (5/44) had 4. In addition, the cohort showed the typical features of an early-onset T1D, such as a high frequency of multiple pancreatic autoimmunity and positive IAA in most cases. Slightly more than half of the patients had DKA at diagnosis and only 4% of patients had a low-risk HLA-DRB1 genotype (Table 1).

### Genetic Analyses

A customized NGS panel of 69 genes related with carbohydrate metabolism disorders was tested in 44 patients with T1D onset between 9 and 30 months of age. The genetic screening identified 4 rare variants (MAF < 0.01) in heterozygous state in genes known to cause monogenic autoimmune diabetes: 2 likely pathogenic variants in *STAT5B* and *FOXP3* (patients 1 and 2) and 2 VUS in *LRBA* and *AIRE* (patients 3 and 4) (Table 3). Analysis of the remaining genes associated with nonautoimmune monogenic diabetes did not find any additional variants with clinical significance.

**Table 3.** Likely pathogenic and VUS variants found in genes associated with monogenic autoimmune diabetes

ID	Gene	Location	Nucleotide change	Amino Acid change	Genetic state	Variant Type	MAF	dbSNP	ACMG
**1**	*STAT5B*	Intron 3 -Exon 4	c.286-12_290del	p.(?)	Het	Splice-site deletion	0	None	LPAT
**2**	*FOXP3*	Intron 1	c.-22-2delA	p.(?)	Hemi	Splice-site deletion	<0.0001	rs1557116776	LPAT
**3**	*LRBA*	Exon 5	c.607C>T	p.(Pro203Ser)	Het	Missense	0	None	VUS
**4**	*AIRE*	Exon 6	c.718G>C	p.(Gly240Arg)	Het	Missense	0	None	VUS

Reference sequences: *AIRE* (NM_000383.4), *FOXP3* (NM_014009.4), *LRBA* (NM_001199282.2), STAT5B (NM_012448.4).

Abbreviations: ACMG, American College of Medical Genetics; dbSNP, Single Nucleotide Polymorphism Database; Hemi, hemizygous; Het, heterozygous; LPAT, likely pathogenic; MAF, minor allele frequency from GnomAD_ALL; VUS, variant of unknown significance.

Patient 1 was a boy who developed T1D at 12 months of age with positive IAA, GADA, and IA2A and without family history of diabetes. Genetic testing revealed a heterozygous deletion in *STAT5B* involving a 17-nucleotide deletion from position 286-12 of intron 3 to position 290 of exon 4 (c.286-12_290del). This variant is not found in population databases and has not been described in the literature. Different prediction software suggest a loss of the splicing acceptor site in intron 3 and parental testing confirmed that it was a de novo variant.

Patient 2 was a boy diagnosed with T1D at 9 months of age with positive IAA. Genetic study in this case showed a hemizygous one-nucleotide deletion located in the splicing acceptor site of the 5-UTR region in *FOXP3* gene (c.-22-2delA). This variant has not been previously described in the literature, but it is reported in population databases as gnomAD with an extremely low allele frequency of 1/92378. In addition, predictive tools of pathogenicity indicate a loss of the splicing acceptor site in intron 1. The patient inherited this variant from his mother, a heterozygous carrier without clinical symptoms of diabetes, except for gestational diabetes that subsided after pregnancy.

Patient 3 was a girl diagnosed with T1D at 24 months of age with positive IAA. Genetic testing resulted in a heterozygous missense variant in exon 5 of *LRBA* gene [c.607C>T, p.(Pro203Ser)]. This variant is not found in population databases, nor is there any clinical evidence in the literature on its pathogenicity and prediction software give contradictory results. Parental testing revealed that the variant is inherited from her father, who does not suffer from diabetes.

Patient 4 was a boy diagnosed with T1D at 18 months of age. Autoantibody testing revealed IAA, IA2A, and ZnT8A positivity and genetic testing identified a heterozygous missense variant in exon 6 of *AIRE* [c.718G>C; p.(Gly240Arg)]. This variant is not found in population databases, nor is there any clinical evidence in the literature on its pathogenicity and prediction software give contradictory results. Parental testing revealed that the variant is inherited from his father, who has no symptoms of diabetes.

### Impact on mRNA Splicing

To know the impact of the *STAT5B* and *FOXP3* variants on mRNA splicing, we performed RNA analysis by RT-PCR and subsequent sequencing. The results are detailed as follows.

**Figure 1.**
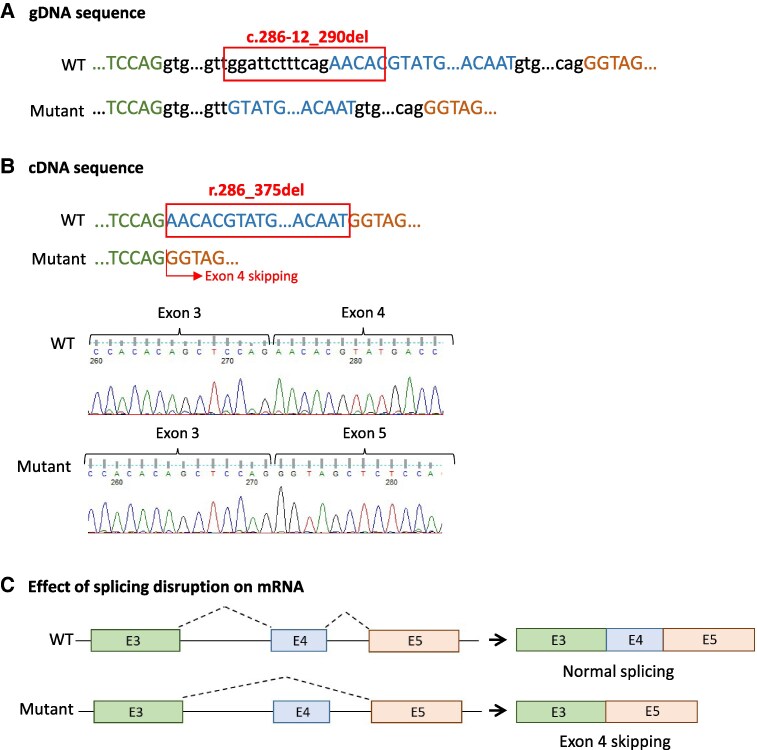
RNA analysis of the STAT5B splice deletion in patient 1 by RT-PCR and sequencing. (A) gDNA sequences showing the position of the STAT5B deletion found (NM_012448.4:c.286-12_290del) in the wild-type (WT) and in the mutant allele. (B) cDNA sequence and Sanger chromatogram of the STAT5B in the WT and the mutant allele. (C) Schematic representation of the impact of the splice-site deletion on mRNA. The variant c.286-12_290del results in exon 4 skipping at cDNA level (r.286_375del). Boxes and lines symbolize exons and introns, respectively.

The human *STAT5B* gene (NM_012448.4) mapped on 17q11.2 comprises 19 exons encoding a protein of 787 amino acids that consists of several domains: the amino-terminal, the coiled-coil, the DNA-binding, the linker, the Src homology 2 (SH2), and the transactivation domains. RT-PCR and subsequent sequencing demonstrated that *STAT5B* splice site variant allele (c.286-12_290del) results in abnormal splicing leading to a truncated transcript missing the entire exon 4 (r.286_375del) (Fig. 1). This exon 4 skipping leads to an in-frame deletion of 30 amino acids (NP_036580.2: p.Asn96_Asn125del) located at the amino-terminal domain, which is an important site for protein-protein interactions, influencing the subsequent signaling cascade and thus the correct *STAT5B* function (23-25).

The *FOXP3* gene (NM_014009.4), mapped to Xp11.23, comprises 12 exons. The noncoding exon 1 corresponds entirely to the 5-UTR, interrupted by a 6011-bp intron 1 that contains a splice site 22 bp upstream of the translation start site (26, 27).

**Figure 2.**
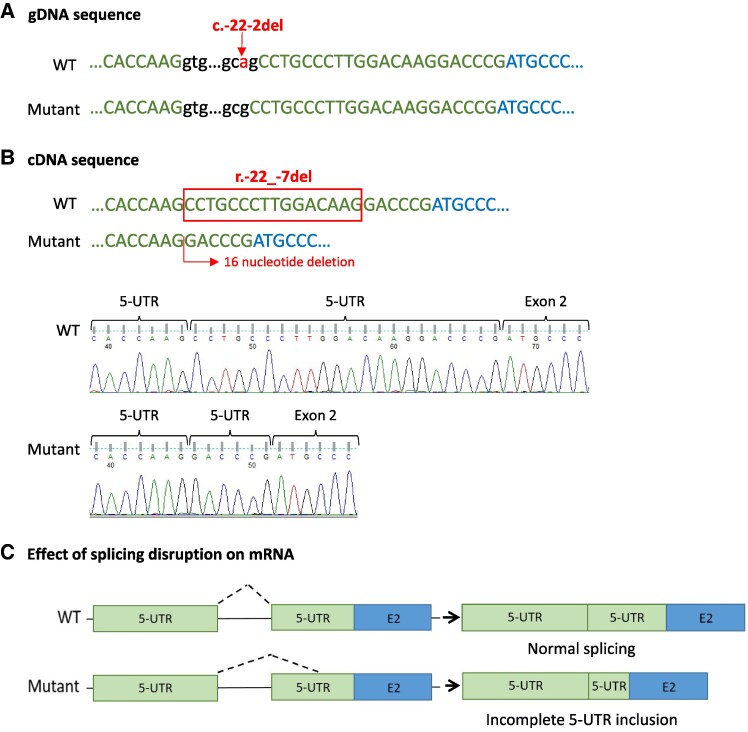
RNA analysis of the FOXP3 splice deletion in patient 2 by RT-PCR and sequencing. (A) gDNA sequences showing the position of the FOXP3 splice site deletion found (NM_014009.4:c.-22-2del) in the wild-type (WT) and in the mutant allele. (B) cDNA sequence and Sanger chromatogram of FOXP3 in the WT and the mutant allele. (C) Schematic representation of the impact of the splice-site deletion on mRNA. The variant (c.-22-2del) results in the deletion of 16 nucleotides (r.-22_-7del) of the 5-UTR region just upstream of the transcription start site. Boxes and lines symbolize exons and introns, respectively.

The *FOXP3* RNA analysis revealed that the intronic deletion of a single adenine located in a highly conserved acceptor site in the intron 1 of the *FOXP3* gene (c.-22-2del), affects the mRNA splicing. cDNA sequencing showed a 16 nucleotide deletion in the 5-UTR (r.-22_-7del), before the transcription start (Fig. 2). Despite being outside coding regions this deletion could interfere with gene expression because of the presence in this area of conserved noncoding sequences responsible of protein regulation (27).

## Discussion

Monogenic forms of autoimmune diabetes share clinical features with T1D, such as an early onset, the presence of pancreatic autoimmunity, or a favorable response to treatment with exogenous insulin. Additionally, the immunological phenotype of this disorder is heterogeneous, and many patients show atypical features with a milder clinical course, giving the possibility of being misdiagnosed as T1D, as has been demonstrated with nonautoimmune monogenic diabetes in pediatrics (14, 28). All this suggests that a monogenic form of autoimmune diabetes could go unnoticed among children with an unusual early T1D onset before the age of 30 months. Following this hypothesis, we identified 2 novel likely pathogenic variants affecting canonical splicing sites in 2 genes associated with monogenic forms of autoimmune diabetes: c.286-12_290del in *STAT5B* and c.-22-2del in *FOXP3* (patients 1 and 2, respectively).

Mutations in the signal transducer and activator of transcription (STAT) protein family result in primary immunodeficiencies and other immune dysregulation syndromes, which are characterized by autoimmunity that often coexists with T1D (29). In this study, RNA analysis confirms that the novel variant in *STAT5B* gene is responsible for exon 4 skipping, which is highly expected to affect the correct function of *STAT5B*. Despite being poorly documented in humans (7, 30), a correlation of *STAT5B* deficiency with pathogenesis of diabetes in animal models is well established (31), supporting the possibility that the variant is responsible for the T1D in the patient. In addition, although most of the loss-of-function mutations in *STAT5B* described so far are autosomal recessive and associated with GH insensitivity syndrome with immune dysregulation type 1 (23), dominant mutations have also been described that have milder effects on IGF-I expression, growth, and immune complications (32). Therefore, the likely pathogenic variant in heterozygosis in *STATB5* could be responsible for the autoimmune diabetes in the patient.

The second likely pathogenic variant detected by our screening corresponds to the *FOXP3* gene located in chromosome X. This gene is highly conserved across mammals and encodes a key transcription factor required for regulatory T-cell development, maintenance, and function, which is essential for preserving immune tolerance (33). In males, hemizygous loss-of-function mutations in this gene cause immune dysregulation, polyendocrinopathy, enteropathy, X-linked (IPEX) syndrome mainly characterized by severe enteropathy, chronic dermatitis, and early-onset autoimmune diabetes, in addition to other possible autoimmune conditions (34). However, this classic disease pattern does not always manifest itself, and patients with atypical features or with a milder clinical course are increasingly being reported (35, 36). The RNA analysis performed in this study demonstrates that the adenine deletion located in the splicing acceptor site of the 5-UTR region of the *FOXP3* gene (c.-22-2del) affects mRNA splicing, triggering a 16-nucleotide deletion in the mRNA region just upstream of the transcription start site. There is evidence in the literature of pathogenic variants in the 5-UTR that can influence translation efficiency and often result in altered protein synthesis by disrupting the regulation of expression attributed to this highly structured region (37). This is the case for other previously described pathogenic variants in *FOXP3* that affect the first splice donor site in the 5-UTR region leading to an atypical and attenuated phenotype of IPEX syndrome (38). In addition, other patients with *FOXP3* mutations have also been described with early-onset autoimmune diabetes with hardly any other clinical manifestations (10). Therefore, all evidence strongly suggests that this alteration is responsible for autoimmune diabetes in the patient even if he does not present other autoimmune conditions at this time.

Both patients with likely pathogenic variants had T1D risk HLA-DRB1 genotypes: moderate risk for patient 1 and high risk for patient 2, which could suggest a diagnosis of T1D. However, although it is well known that there are HLA haplotypes that clearly predispose to T1D, pathogenic variants in autoimmunity-related genes cannot be excluded in these particular cases because they could contribute to diabetes development at an earlier age than would be expected for classic T1D. In fact, recent studies highlight shared pathways for the pathogenesis of polygenic and monogenic autoimmune diabetes (5). Moreover, the high-risk T1D DR3-DQ2 haplotype has been shown to contribute to diabetes development in monogenic autoimmunity (39). Further molecular characterization of the mechanisms responsible for both the monogenic and polygenic forms of the disease will be essential for detecting the pathways underlying this shared etiology and will allow the development of appropriate therapies (5, 8).

We have also found 2 VUS variants in heterozygosis in *LRBA* and *AIRE*. Unfortunately, there is insufficient evidence to determine whether they are related to the disease or have any clinical significance in the affected patients. Therefore, the molecular genetic screening performed in our cohort showed a suspected monogenic form of autoimmune diabetes in at least 4.5% (2/44) of pediatric patients who were diagnosed with T1D between 9 and 30 months of age. However, previously published studies aimed at screening for monogenic autoimmune diabetes among pediatric patients with T1D, find a 5 to 10 times higher percentage than in our cohort (6, 9). This difference can certainly be explained by the type of patient recruitment because both studies cited focus on patients with a more severe and syndromic clinical picture, which considerably increases the likelihood of patients suffering from a rare type of monogenic autoimmune diabetes. On the other hand, in a previous study that analyzed monogenic diabetes in 64 patients diagnosed before 1 year of age (40), a mutation causing monogenic autoimmunity was found in 2 patients, 1 in *STAT3* and the other in *FOXP3*. In our cohort, the 2 patients with suspected monogenic autoimmune diabetes were younger than 1 year old at the time of T1D onset, accounting for a significant 22% (2/9) of patients younger than 1 year with possible unrecognized monogenic autoimmunity.

Suspicion of monogenic autoimmune diabetes is commonly focused on patients with diabetes and other autoimmune conditions. Our study provides a different approach targeting exclusively patients with an early onset of T1D regardless of whether they have other autoimmune disorders. This new approach is the main strength of the study because it alerts us of the possibility of unrecognized monogenic autoimmune diabetes among pediatric patients with T1D. In fact, it is described that autoimmune diabetes may be the first clinical manifestation and that other autoimmune conditions may appear later (5), which could be the case for patients 1 and 2 in this study. Another strength is the NGS approach that extends the search to all major genes currently associated with the disease. However, the possibility of not correctly diagnosing patients with rarer forms of monogenic autoimmune diabetes cannot be completely excluded, as patients may have pathogenic variants in new genes associated with the immune system that are not included in the panel or in unchecked noncoding regions. On the other hand, the genetic study has been carried out together with RNA analyses, which has demonstrated that the 2 new likely pathogenic variants alter mRNA splicing. With this result, it is expected that the variants alter the conformation and, consequently, the function of the protein in the case of *STAT5B*, and the normal expression of the gene in the case of *FOXP3*. These 2 novel variants broaden the spectrum of mutations that can cause monogenic forms of autoimmune diabetes in pediatrics.

In conclusion, this study underlines the convenience of genetic screening in pediatric patients with uncommon features of T1D. With advances in NGS, genetic testing may be indicated in patients with unusual early-onset autoimmune diabetes. At present, these patients are unlikely to undergo genetic testing for monogenic autoimmune etiology in the absence of a suggestive clinical phenotype. However, we recommend molecular testing to at least 1 year of age, as early detection is crucial to guide medical treatment, give insight into prognosis, as well as genetic counseling when appropriate.

## Data Availability

Some or all datasets generated during and/or analyzed during the current study are not publicly available but are available from the corresponding author on reasonable request.

## Abbreviations

DKA: diabetic ketoacidosis
IAA: insulin autoantibodies
GADA: glutamate decarboxylase autoantibodies
IA2A: islet tyrosine phosphatase 2 autoantibodies
IPEX: immune dysregulation, polyendocrinopathy, enteropathy, X-linked
MAF: minor allele frequency
NGS: next-generation sequencing
T1D: type 1 diabetes
5-UTR: 5′ untranslated region
VUS: variant of unknown significance
ZnT8A: zinc transporter-8 autoantibody
